# Mechano-electric feedback effects in a three-dimensional (3D) model of the contracting cardiac ventricle

**DOI:** 10.1371/journal.pone.0191238

**Published:** 2018-01-17

**Authors:** Ani Amar, Sharon Zlochiver, Ofer Barnea

**Affiliations:** Department of Biomedical Engineering, Faculty of Engineering, Tel Aviv University, Tel Aviv, Israel; University of Minnesota, UNITED STATES

## Abstract

Mechano-electric feedback affects the electrophysiological and mechanical function of the heart and the cellular, tissue, and organ properties. To determine the main factors that contribute to this effect, this study investigated the changes in the action potential characteristics of the ventricle during contraction. A model of stretch-activated channels was incorporated into a three-dimensional multiscale model of the contracting ventricle to assess the effect of different preload lengths on the electrophysiological behavior. The model describes the initiation and propagation of the electrical impulse, as well as the passive (stretch) and active (contraction) changes in the cardiac mechanics. Simulations were performed to quantify the relationship between the cellular activation and recovery patterns as well as the action potential durations at different preload lengths in normal and heart failure pathological conditions. The simulation results showed that heart failure significantly affected the excitation propagation parameters compared to normal condition. The results showed that the mechano-electrical feedback effects appear to be most important in failing hearts with low ejection fraction.

## Introduction

Investigating the effects of intracellular electromechanical coupling and mechano-electric feedback (MEF) on myocardial function and its contribution to arrhythmogenesis is of great importance. Changes in the electrophysiological behavior explained by stretch-activated channels (SACs) were observed experimentally [1–3]. The impact of mechanical events on cardiac electrophysiology was studied in several cellular and tissue models [4–7] by including stretch activation of ion channels and mechanical modulation of cellular Ca^2+^ handling as the major mechanisms of the MEF. Several strongly coupled electromechanical models of the ventricles described mechanical deformation triggered by electrical activation with consideration of the MEF [8–11]. Such studies typically demonstrate the effect of mechano-electric coupling via the SACs in terms of arrhythmogenesis without taking into account load induced electrophysiological changes. However, the alterations of ventricular loading conditions may provide a basis for initiation of arrhythmia as observed in experimental studies [12,13].

The purpose of this study was to investigate the effects of mechano-electric coupling via stretch-activated ion channels in the contracting ventricle. A previously developed three-dimensional (3D) multiscale model of the contracting ventricle [14] was used to determine the influence of mechanical perturbations on electrophysiological parameters. Thus, an investigation of the effects of mechano-electric coupling was possible that could not be assessed experimentally. The model includes a considerable level of physiological detail and a non-continuum method to represent the myocardium.

The electrophysiological effects of myocardial stretching were evaluated in the contracting ventricle model by varying the preload length and quantitatively analyzing the changes in action potential durations, activation, and recovery patterns. The model describes the initiation and propagation of the electrical impulse and the passive (stretch) and active (contraction) changes in cardiac mechanics. In addition, the model was used to evaluate the effects of different preload lengths on the cardiac electrophysiology under normal and heart failure (HF) pathological conditions.

## Materials and methods

**Fig 1:**
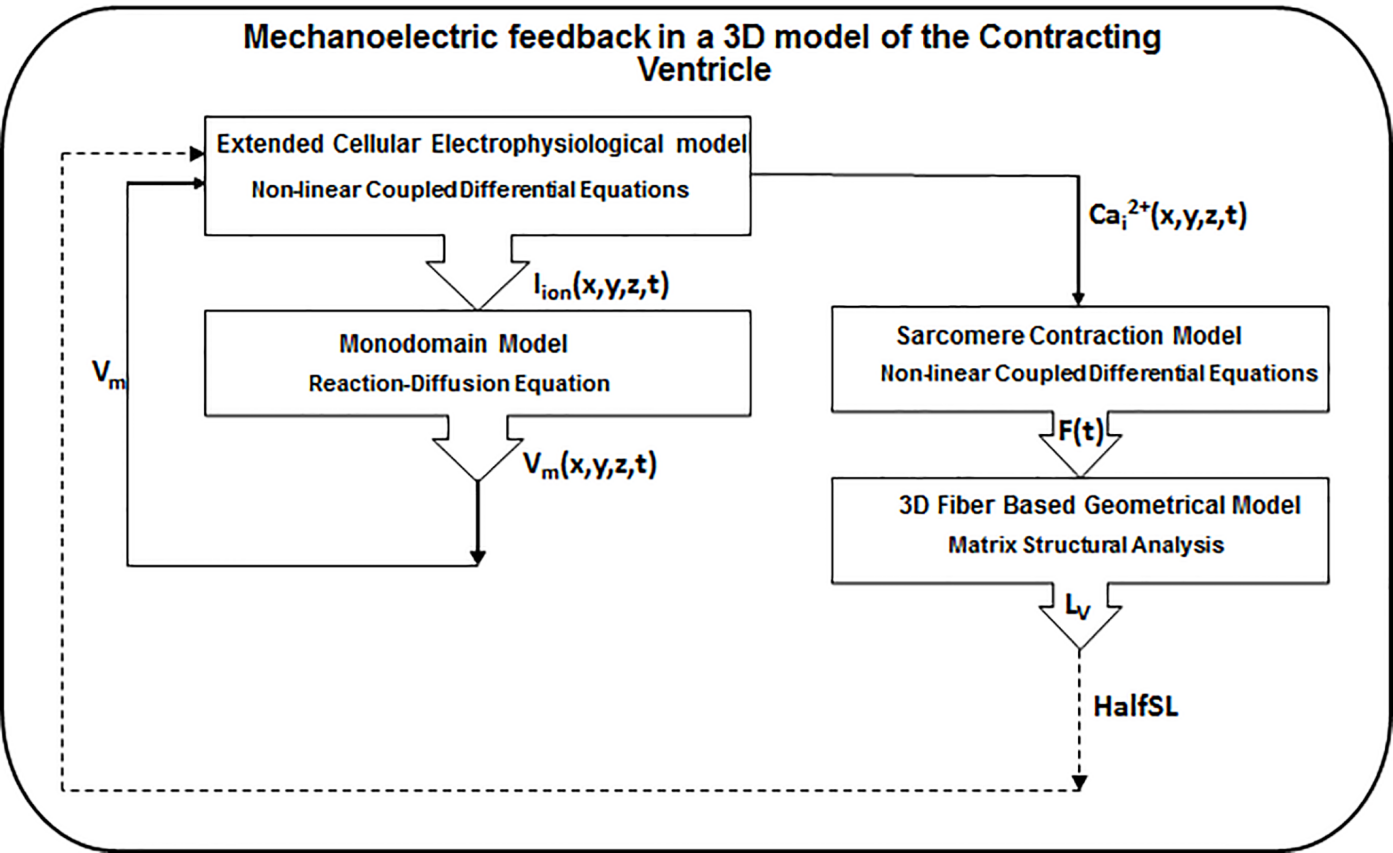
Flowchart of the MEF simulation in the 3D model of the contracting ventricle. The dashed arrow shows the implementation of mechanical feedback in the model.

The simulation of MEF in this study consists of four major components: the cellular electrophysiology, force development, excitation propagation, and anatomical structure (Fig 1). These components were combined to form the 3D multiscale ventricular model for evaluating the influence of initial length of cardiac tissue (preload) on cardiac electrophysiology. The 3D model of the contracting ventricle was described earlier [14]. In this study, a brief overview of the contracting ventricle model was provided.

### Electrophysiological behavior

The electrophysiological model was based on the ten Tusscher-Noble-Noble-Panfilov (TNNP) heterogeneous model of a human ventricular myocyte [15]. It described 12 ionic currents, variations in the intracellular concentration of Na^+^, K^+^, and Ca^2+^ ions, and the transmembrane voltage. The time-dependent changes in the transmembrane voltage *V*_*m*_ [mV] were described by the following differential equation:

$$\frac{\partial V_{m}}{\partial t}=-\frac{1}{C_{m}}\left(I_{ion}-I_{stim}\right)$$ (1)

where *t* [ms] was the time, and *C*_*m*_ [*μ*F/cm^2^] was the cell capacitance per unit surface area. The parameter *I*_*ion*_ [*μ*A/*μ*F] was the sum of the membrane ionic currents through the ionic channels located on the sarcolemma, and *I*_*stim*_ [*μ*A/ *μ*F] was the external stimulus current. In this study, thet TNNP model was extended by adding a stretch-activated channel (*I*_*SAC*_) that corresponded to the stretch effects on the action potential (AP). The following sections describe the details. The sum of the transmembrane ionic currents in the electrophysiological model was described by *Eq 2*.

$$\begin{array}{l} I_{ion}=I_{Na}+I_{NaK}+I_{bNa}\\ +I_{K1}+I_{to}+I_{Kr}+I_{Ks}+I_{pK}\\ +I_{CaL}+I_{NaCa}+I_{pCa}+I_{bCa}\\ +I_{SAC} \end{array}$$ (2)

The fast Na^+^ current was *I*_Na_. The Na^+^/K^+^ pump current was *I*_NaK_, and the background Na^+^ current was *I*_bNa_. The parameter *I*_K1_ was the inward rectifier K^+^ current, and the transient outward current was *I*_to_. The rapid delayed rectifier current was *I*_Kr_, and the parameter *I*_Ks_ was the slow delayed rectifier current. The plateau K^+^ current was *I*_pK_, and the L-type Ca^2+^ current was *I*_CaL_. The parameter *I*_NaCa_ was the Na^+^/Ca^2+^ exchanger, and the plateau Ca^2+^ current was *I*_pCa_. The background Ca^2+^ leak current was *I*_bCa_., For all simulations the TNNP model was applied with the parameter set of a human mid-myocardial ventricular cell. *Eq 1* was solved using the Euler's method with a time step of Δt = 0.02 ms. This time step ensured a stable converging solution for the TNNP model equations.

### Heart failure

To represent electrophysiological changes due to HF, the modifications proposed by Zlochiver were incorporated [16]. Briefly, HF condition in the model was introduced by varying maximum conductances of the transient outward current (*I*_to_*)*, the slow delayed rectifier current (*I*_Ks_*)*, the inward rectifier K^+^ current (*I*_K1_), the fast Na^+^ current (*I*_Na_), the Na^+^/Ca^2+^ exchanger (*I*_NaCa_) and L-type Ca^2+^ current (*I*_CaL_) with values listed in S1 Table in the Supporting information.

In the model, the propagation of electrical excitation was represented by the reaction-diffusion equation for the isotropic medium, as described previously [14].

### Mechano-electricfeedback

#### Stretch-activated channels

The effect of stretch-activated channels on the cellular electrophysiological state was implemented by integrating the formulation of the SACs into the electrophysiological model based on the formulation by Zabel et al. [2] with some modifications.

$$I_{SAC}=G_{SAC}\times \frac{V_{m}-V_{rev}}{1+K\cdot e^{-\alpha \left(\lambda -1\right)}}$$ (3)

The parameter *V*_*m*_ [mV] was the transmembrane voltage, and *V*_*rev*_ [mV] was the SAC reversal potential. The parameter *G*_*SAC*_ [nS/pF] was the maximum SAC conductance, the parameter *K* defined the amount of current when the cell was not stretched, and *α* was the parameter that controlled the sensitivity to stretch. The sarcomere length in *Eq 3* was replaced by the stretch ratio *λ* that was calculated as the ratio between the current and the resting half sarcomere length (0.97 μm) obtained from the myofilament model [17]:

$$\lambda =\frac{HalfSL}{HalfSL_{0}}$$ (4)

All the parameter values discussed above are listed in Table 1. Due to the absence of consistent data on the SACs, a reversal potential *V*_*rev*_ of -20 mV [2] and a maximum conductance *G*_*SAC*_ of 0.025 nS/pF (a similar range was observed by Sachs et al. [18]) were chosen.

**Table 1:** Numerical values of the parameters in the SAC formulation.

Parameter	Value	Units	Source
*HalfSL*_*0*_	0.97	μm	[17]
*K*	1	[]	model fit
*α*	3	[]	[2]
*E*_*SAC*_	-20	mV	[2],[18]
*G*_*SAC*_	0.025	nS/pF	[18]

### Force development model

The intracellular calcium released during an electrical activation coupled the electrical and mechanical components in the 3D model simulations. This [Ca^2+^]_*i*_ was used as an input parameter for the myofilament model of Negroni and Lascano (NL) [17] previously described [14]. The NL model was incorporated into the simulation to represent the force development and sarcomere shortening within each myocyte.

### Fiber-based geometrical model

The fiber-based model of the contracting ventricle [19] formed the geometrical basis for the evaluation of the MEF effects. The 3D structure of the model and the modeling method of the cardiac mechanics were described in earlier research [14]. A twitch obtained from the sarcomere model of force generation [17] (described in the previous section) was used as an input function for the simulation of twitch propagation in the model.

#### Mechanical deformation

**Fig 2:**
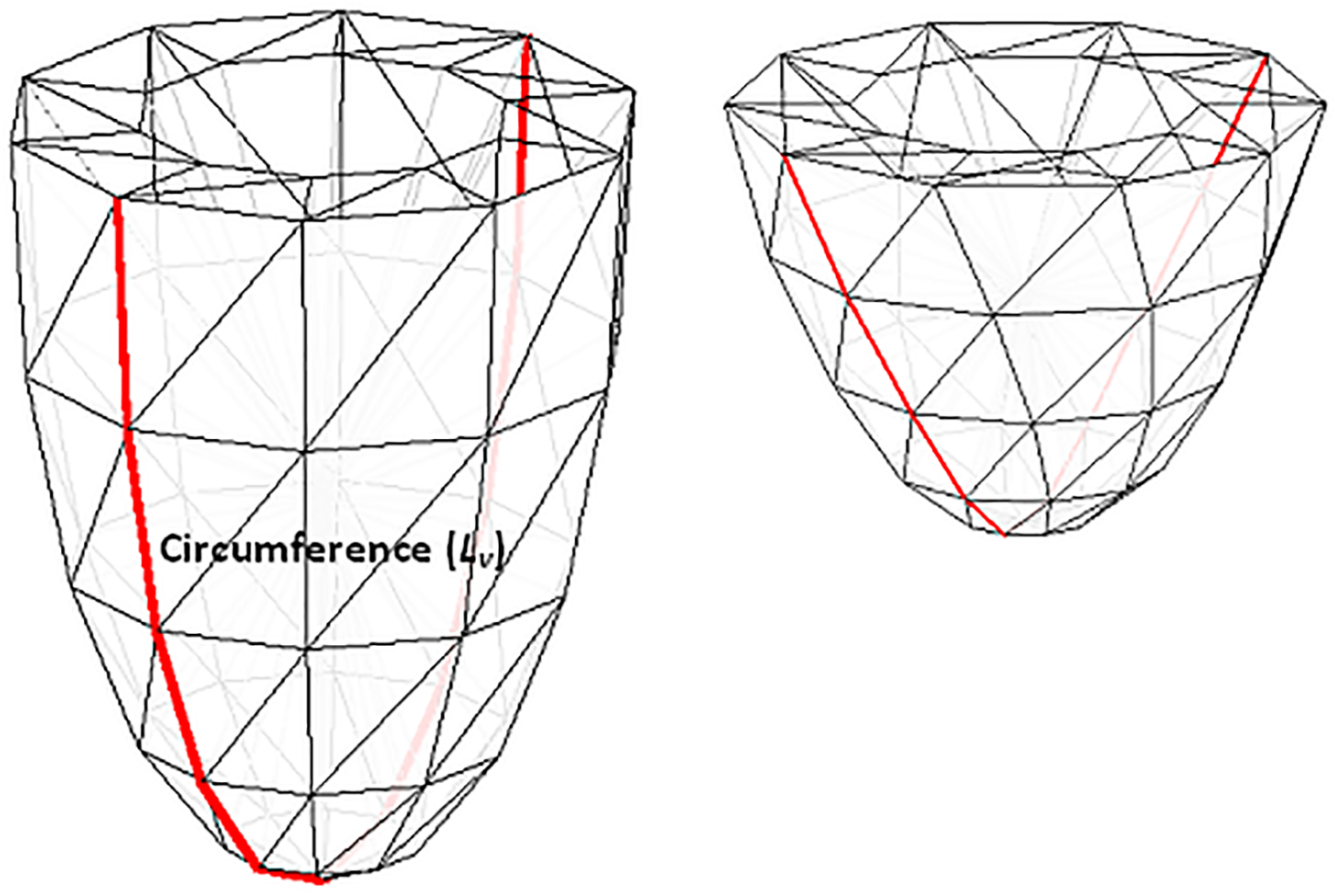
**3D fiber-based geometrical model of the contracting ventricle (a) before and (b) after contraction.** The *L*_*V*_ (red line) denotes the outer boundary (circumference) of the ventricle.

The layer of the ventricle was described by the number of muscle units connected in series. As mentioned previously [14], the volume of the ventricle model was estimated for each time step of the analysis. Thus, the length of the outer boundaries *L*_*V*_ (circumference) of the ventricle (Fig 2) could be calculated. Using the approach of Shim et. al [20], changes in the volume of the ventricles could be translated into the changes in the half- sarcomere length by dividing the length *L*_*V*_ by the number of half sarcomeres (*N*) in the unstressed ventricle:

HalfSL=LVN (5)

The updated half-sarcomere length was used to calculate the *I*_*SAC*_ current that was added to the TNNP electrophysiological model and, in turn affected the AP characteristics.

### Simulation protocol

Several sets of simulations were performed to compare the electrophysiological behavior of the stretched tissue with the SACs recruitment. The first simulation runs were performed to investigate the influence of the SACs on the AP at the single cell level by applying a steady stretch. The cell was excited by applying a stimulus current (52 pA·pF^-1^) at t = 0 ms with a length of 1 ms. The stretch was induced by changing the resting half-sarcomere length. The effects of the stretch were simulated when the cell was not stretched (λ = 1) and at different degrees of stretch (λ = 1.10, 1.20, 1.25, and 1.30).

The second simulation was performed with the 3D model of the contracting ventricle. In these simulations, the AP propagation was compared in the tissue with different half-sarcomere length values (0, 10, 15, 20, and 25% of the reference length) and associated preload values. The stimulus was applied for 6 ms at the predetermined element of the ventricle apex. The local activation time (AT) was determined as the time when the membrane potential upstroke reached -60 mV. The local recovery time (RT) was defined as the time when the repolarized membrane potential reached -60 mV (typically 80% of repolarization). The duration of the AP (APD) of each individual myocardial fiber was calculated as a time interval between the initial activation and recovery of the fiber.

For the next simulations HF pathological condition was incorporated into the TNNP model (a detailed description of the integrated modifications is presented in S1 Table in the Supporting information). The cell was excited under the same conditions as those in the first simulation. No stretch was applied during these simulation runs. At the system level, the corresponding pressure-volume (PV) loops were generated with the fixed preload and the control set of the loading parameters (see [14] for details). The excitation propagation in the 3D model of the contracting ventricle under HF condition was carried out in the same manner as the second simulation.

## Results

**Fig 3:**
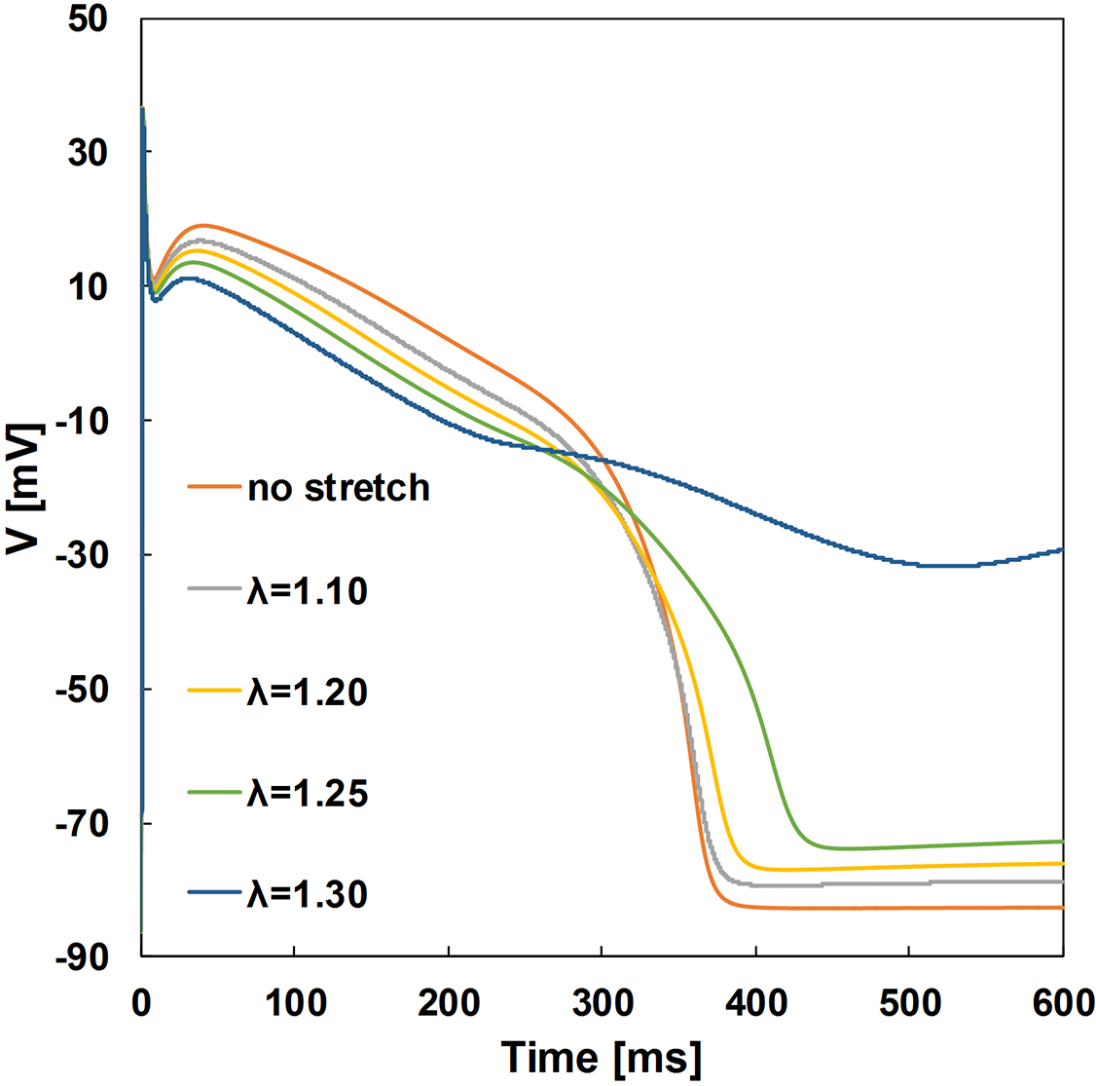
Stretch effects on the simulated APs at different degrees of steady-state stretch (λ = 1.10 to 1.30).

The contribution of the SACs to the stretch effects on the AP is shown in Fig 3. The effects of the steady stretch were simulated at different degrees of the stretch ratio λ. When the stretch increased, the early phase of repolarization of the AP was accelerated, while late repolarization was prolonged for the stretches of 10%, 20%, and 25% (λ = 1.10, 1.20, and 1.25, respectively). A large stretch (30% of the reference length) depolarized the membrane and prevented repolarization of the AP. The stretches of 10–20% led to an increase in the resting potential by approximately 10 mV. The crossover of the repolarized APs occurred near -20 mV. The peaks of the AP amplitudes obtained during the simulations were independent of the stretch.

**Fig 4:**
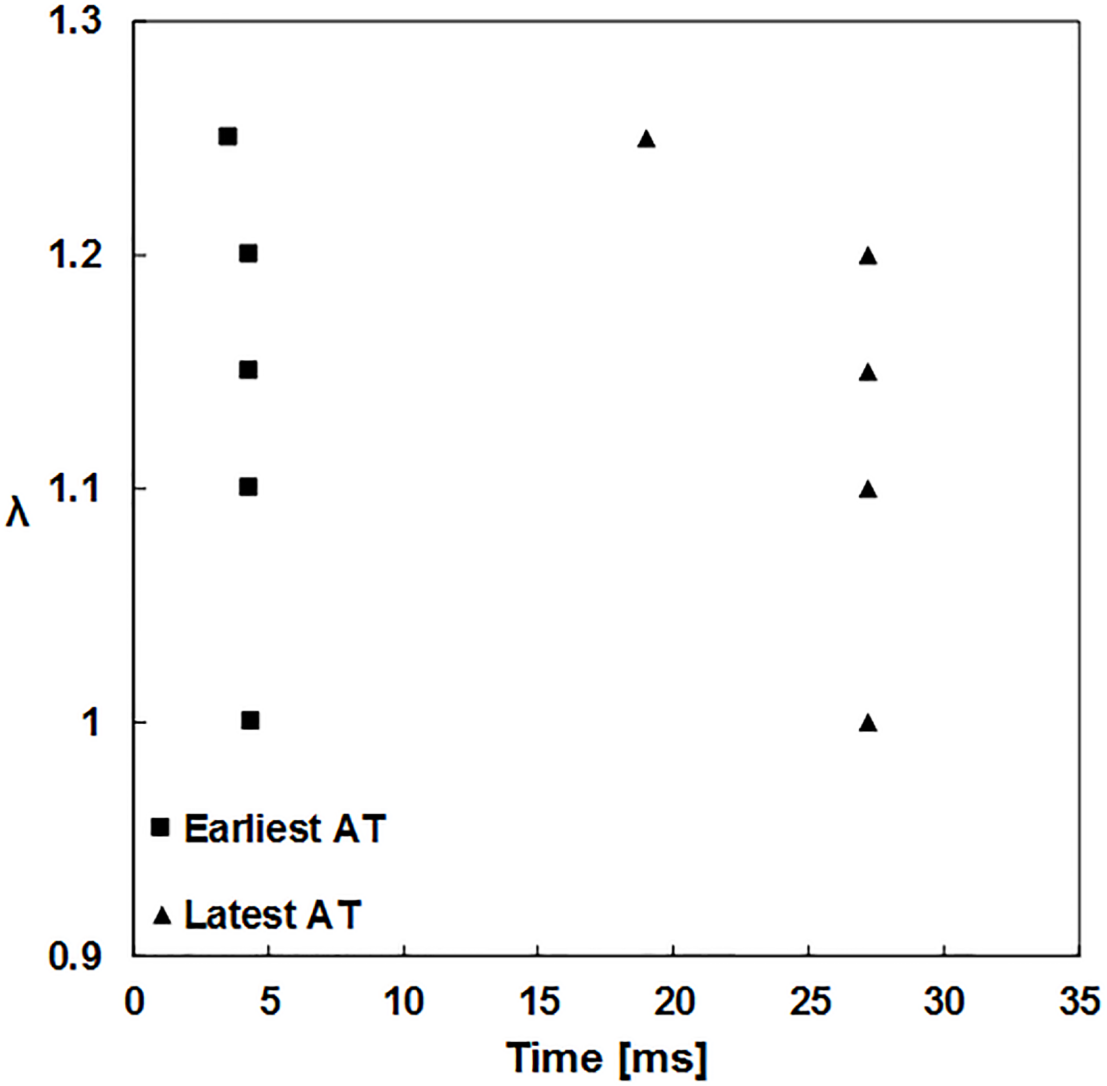
Comparison of the ATs influenced by different preload lengths (stretch ratio λ = 1.00, 1.10, 1.15, 1.20, and 1.25). AT–activation time.

The second simulation examined the effects of different preload lengths on the excitation propagation through recruitment of the SACs. Several AP characteristics were compared following the ventricle stimulation. The first AT for λ = 1 (resting length) was observed at the stimulus site at 4.4 ms (Fig 4). No effect was observed on the earliest ATs (the first ATs compared between the fibers) and the latest ATs the last ATs compared between the fibers) when the preload length increased to 20%. A larger increase (25%) produced an acceleration of the latest AT.

**Fig 5:**
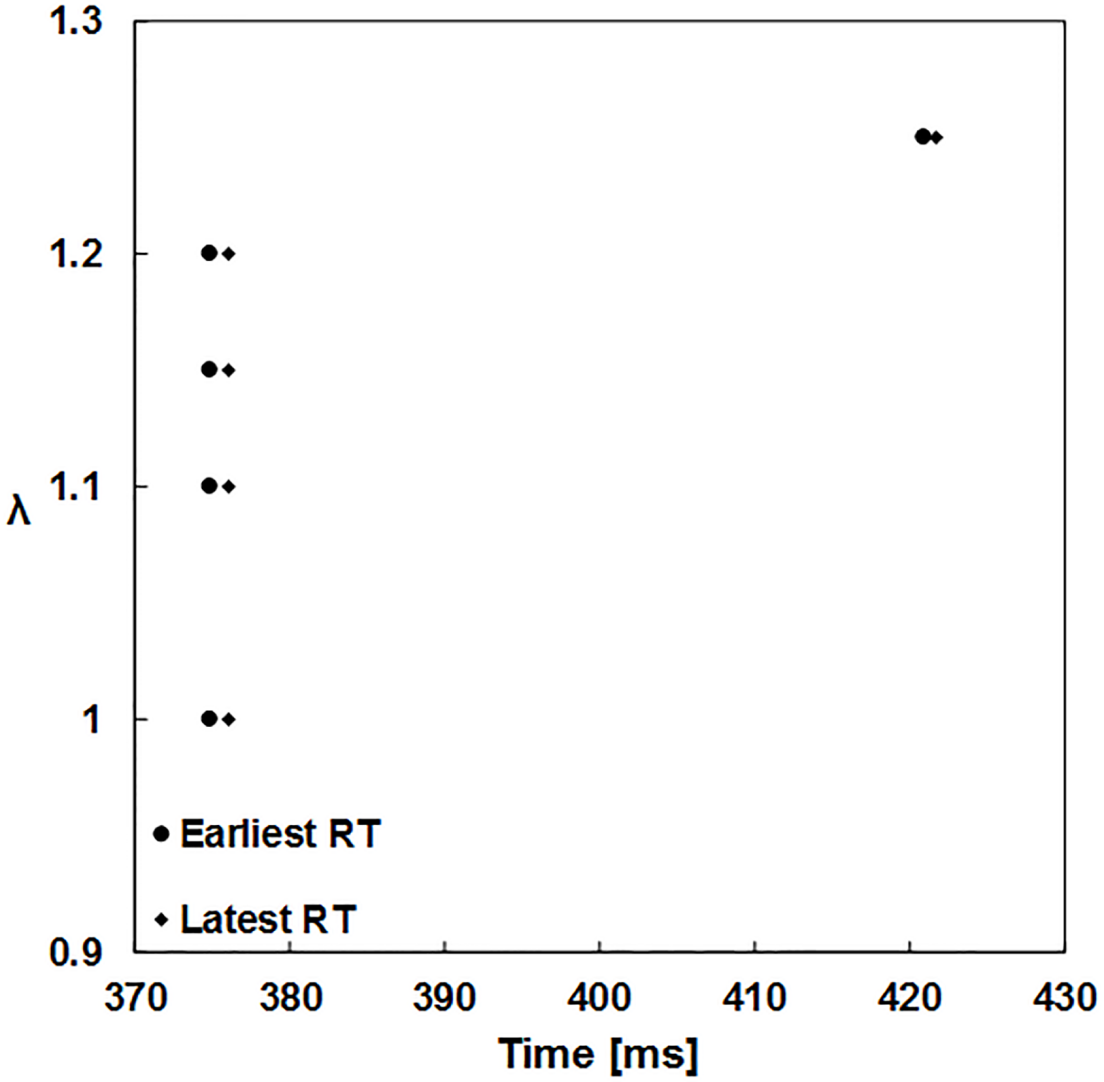
Comparison of the RTs influenced by the different preload lengths (stretch ration λ = 1.00, 1.10, 1.15, 1.20, and 1.25). RT–recovery time.

Fig 5 shows the RTs obtained during the simulation. After the apex stimulation, the first RT occurred at 374.9 ms from the onset of the applied stimulus. Despite the increase of λ, the RTs remained unchanged when the preload length increased by 20%. A 25% increase in the preload length significantly delayed the late repolarization. This compared well with the results obtained in the single cell simulations.

### Excitation propagation during heart failure

The next set of simulations was performed to examine the influence of different preload lengths on electrophysiological parameters under heart failure condition.

**Fig 6:**
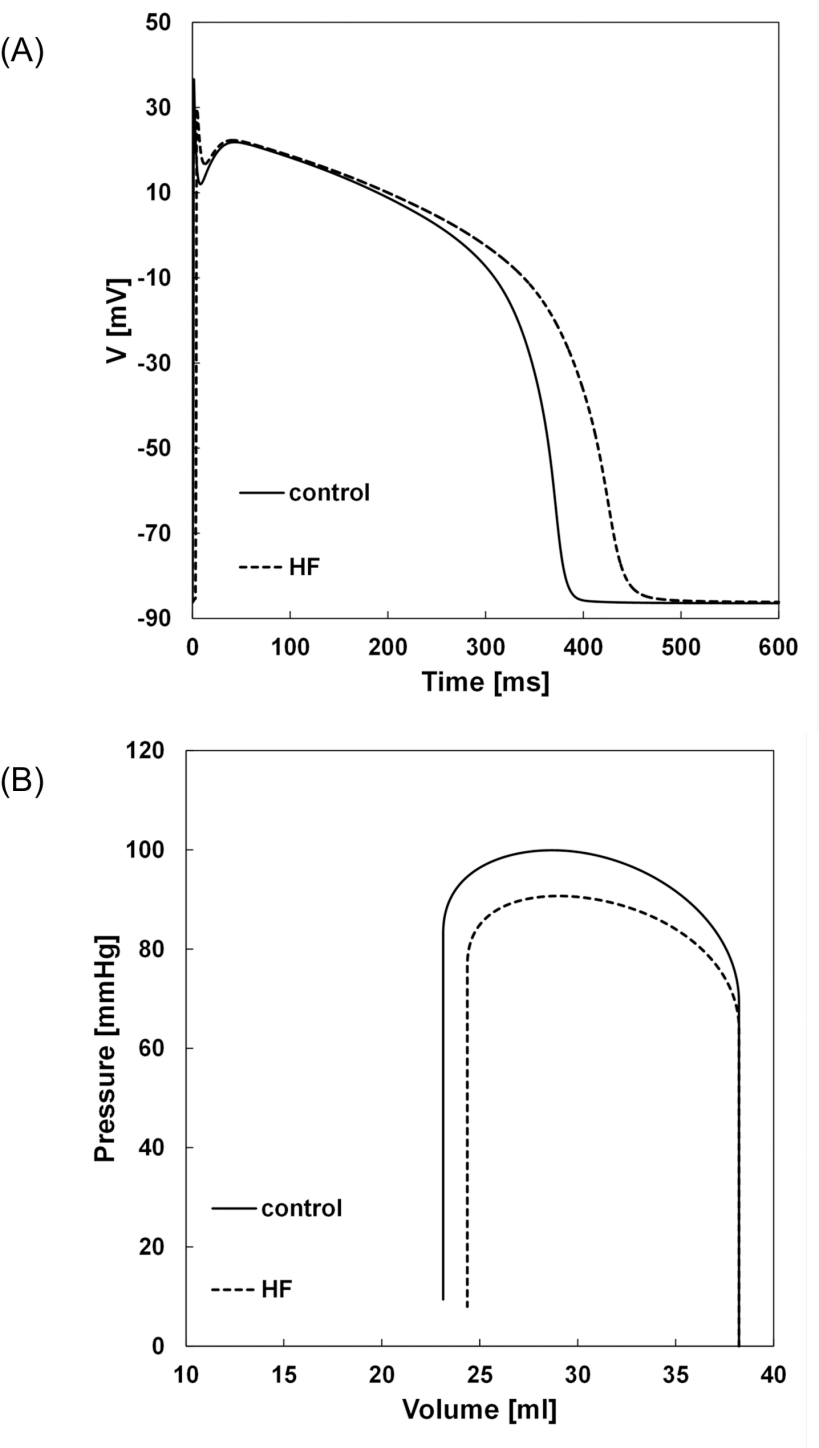
Time course of the simulated APs (A) and the corresponding PV loops (B) under control and HF conditions.

Fig 6A showed the time course of the transmembrane voltage at 1 Hz steady state pacing in control and HF conditions. The figure showed the prolongation of simulated HF action potential compared to control (the original TNNP model). The corresponding PV loops were illustrated in Fig 6B. It can be seen that at a constant preload there was a decrease in ventricular pressure and stroke volume in HF PV loop compared to the normal PV loop.

In order to make comparisons, the APDs generated in the tissue with different preload lengths under normal and HF conditions were analyzed. The calculated APDs are listed in Table 2. The obtained APD_80_ values (the action potential duration at 80% repolarization) for varying preload lengths (0%, 10%, 15%, 20%, and 25% of the reference length) were compared. No changes were found in APDs under control condition, while HF resulted in significantly prolonged APDs for preload lengths up to 20% of the tissue reference length. For a 25% increase in the preload length at control condition, the APD_80_ was increased and was approximately 50 ms longer than that of the smaller length changes. A 25% increase in the preload length at HF prevented repolarization of the APs.

**Table 2:** Comparison of the APD at different degrees of stretch under normal and HF conditions.

Stretch ratio	Min APD (control)	Min APD (HF model)	Max APD (control)	Max APD (HF model)
**λ = 1.00**	348.9	410.5	370.5	434.3
**λ = 1.10**	348.9	445.1	370.6	465.5
**λ = 1.15**	348.9	467.2	370.6	486.8
**λ = 1.20**	348.9	510.9	370.6	529.4
**λ = 1.25**	402.7	N/A^a^	417.4	N/A^a^

^a^ λ = 1.25 prevented the AP repolarization.

## Discussion

In this study, we investigated the effects of mechano-electric coupling in the heart at the system level using a previously developed 3D multiscale model of the contracting ventricle [14]. To understand the effects, the model was modified by adding the SAC that corresponded to the stretch effects on the AP.

The analysis of the MEF effects in the 3D model included simulations with the SACs induced by changing the preload lengths. At the first stage, the effect of a steady stretch on the AP in a single myocyte was assessed. A number of experimental studies have shown that myocardial stretch may shorten or lengthen the APD. A majority of the experiments with rabbit or canine hearts showed a lengthening of the APD during the late repolarization phase [2,12]. Similarly, at a single cell level, this study showed APD shortening at the early repolarization phase and delayed repolarization and crossover of the APs near –20 mV (Fig 3). This was in agreement with the experimental results [2, 21].

The second stage of the simulations evaluated the electrophysiological behavior of the 3D model at the system level for different preload lengths with SAC recruitment. The activation and recovery times as well as the minimum and maximum APD for varying preload lengths (0%, 10%, 15%, 20%, and 25%) were estimated and compared. Hansen et al. [22] showed a shortening of the plateau phase of the AP and a delay during phase 3 repolarization owing to the mechanical stretch that occurred from an increase of the ventricular preload. In this study, a 10–20% increase in the preload length had no effect on the AT and RT during excitation propagation. However, the recovery patterns were affected significantly by a 25% increase in the preload length of the tissue reference length. This was in agreement with the results of Hansen et al. [22]. In contrast to the single cell results, during the system level simulations, an increase in the preload length of 20% did not influence the APD_80_ values. However, for a 25% increase, the APD80 was enlarged. Similar observations were obtained by Hansen [22], where minor changes in the measured APDs at 90% repolarization while altering the preload values were reported.

The model was used then to assess the influence of different preload lengths on electrophysiological parameters under HF condition. HF was modeled by decreasing the maximum conductance of the potassium currents (*I*_to_, *I*_Ks_, *I*_K1_), the sodium current *I*_Na_ and the calcium current *I*_CaL_ while increasing the Na^+^/Ca^2+^ exchanger–the mechanisms that contributed to AP prolongation [23]. The simulated action potentials of normal and failing ventricular cells were of similar morphology to measured physiological data (Fig 6A). The corresponding PV loops (Fig 6B) demonstrate an expected decrease in inotropy (less ventricular pressure was generated and stroke volume was decreased) as was observed experimentally [24]. These simulation results showed that the model presented realistic ventricular contractile performance under HF condition.

Results of the subsequent simulations showed that the presence of heart failure had significant effect on excitation propagation. The ATs and RTs, the minimum and maximum APDs for varying preload lengths to 20% were affected. It can be seen that the mechano-electrical feedback effects that we observed appear to be most important in failing hearts with low ejection fraction. When the preload length was above 20% of the tissue reference length, it depolarized the membrane and prevented repolarization of the APs. These results reflect the realistic mechanisms of heart where the changes above 20% are physiologically irrelevant. Finally, the simulation findings of this study can be interpreted within the framework of the stretch-activated channel hypothesis for further investigation of the influence of mechanical perturbations on electrophysiological parameters.

### Limitations

To ensure the usefulness of the developed model, a number of assumptions were made during design. As there was just one layer of myocardial fibers in the 3D model, transmural heterogeneity was not incorporated in this study. This could potentially affect the activation pattern. Another limitation refers to the propagation of electrical excitation which was represented by the reaction-diffusion equation for the isotropic medium. Consideration of the anisotropy in anatomically accurate geometries, such as previously developed anatomical 3D model of the contracting heart [25], will allow to describe physiologically accurate electrical propagation in myocardial tissue. This will be done in future.

Because of the absence of homogeneous data for the SACs (e.g. channel conductance, density, and reversal potential), the parameter values were chosen from different data sources. This may have reduced the accuracy of the model.

Mechanical modulation of cellular Ca^2+^ was not included in the analysis of the MEF effects. The mechanism by which intracellular Ca^2+^ (the affinity of troponin for calcium) is affected by mechanical perturbations, was not within the scope of this study.

## Supporting information

S1 Table Electrophysiological modifications in HF. (DOCX)
