# Supplementary material for: Mechano-electric feedback effects in a three-dimensional (3D) model of the contracting cardiac ventricle
Source: PLoS One. 2018 Jan 17;13(1):e0191238. doi: 10.1371/journal.pone.0191238 (PMC5771591; doi:10.1371/journal.pone.0191238)
Supplement: S1 Table — (DOCX) [file pone.0191238.s001.docx]

**S1 Table.** **Electrophysiological modifications in HF.**

| Parameter | Percentage of change^a^ |
| --- | --- |
| *g*_to_ maximal transient outward current conductance | ↓36% |
| *g*_Ks_, maximal slow delayed rectifier current conductance | ↓50% |
| *g*_K1_, inward rectifier K^+^ current conductance | ↓43% |
| *g*_CaL_ maximal L-type Ca^2+^ current conductance | ↓30% |
| k_Na‏Ca_ , maximal current density of the Na^+^/Ca^2+^ exchanger | ↑36% |
| *g*_Na_ maximal fast Na^+^ current current conductance | ↓34% |

↓ and ↑ denote decrease and increase relative to the original TNNP model

^a^ Data taken from Zlochiver (2010)
